# Allopurinol and oxypurinol differ in their strength and mechanisms of inhibition of xanthine oxidoreductase

**DOI:** 10.1016/j.jbc.2023.105189

**Published:** 2023-08-23

**Authors:** Mai Sekine, Ken Okamoto, Emil F. Pai, Koji Nagata, Kimiyoshi Ichida, Russ Hille, Takeshi Nishino

**Affiliations:** 1 Department of Applied Biological Chemistry, Graduate School of Agricultural and Life Sciences, The University of Tokyo, Bunkyo-ku, Tokyo, Japan; 2 Department of Pathophysiology, Tokyo University of Pharmacy and Life Sciences, Hachioji, Tokyo, Japan; 3 Departments of Biochemistry and Medical Biophysics, University of Toronto, Toronto, Ontario, Canada; 4 Princess Margaret Cancer Centre, Campbell Family Cancer Research Institute, University Health Network, Toronto, Ontario, Canada; 5 Department of Biochemistry, University of California, Riverside, California, USA

**Keywords:** oxypurinol, allopurinol, xanthine oxidase, xanthine oxidoreductase, gout

## Abstract

Xanthine oxidoreductase is a metalloenzyme that catalyzes the final steps in purine metabolism by converting hypoxanthine to xanthine and then uric acid. Allopurinol, an analog of hypoxanthine, is widely used as an antigout drug, as xanthine oxidoreductase-mediated metabolism of allopurinol to oxypurinol leads to oxypurinol rotation in the enzyme active site and reduction of the molybdenum Mo(VI) active center to Mo(IV), inhibiting subsequent urate production. However, when oxypurinol is administered directly to a mouse model of hyperuricemia, it yields a weaker urate-lowering effect than allopurinol. To better understand its mechanism of inhibition and inform patient dosing strategies, we performed kinetic and structural analyses of the inhibitory activity of oxypurinol. Our results demonstrated that oxypurinol was less effective than allopurinol both *in vivo* and *in vitro*. We show that upon reoxidation to Mo(VI), oxypurinol binding is greatly weakened, and reduction by xanthine, hypoxanthine, or allopurinol is required for reformation of the inhibitor-enzyme complex. In addition, we show oxypurinol only weakly inhibits the conversion of hypoxanthine to xanthine and is therefore unlikely to affect the feedback inhibition of *de novo* purine synthesis. Furthermore, we observed weak allosteric inhibition of purine nucleoside phosphorylase by oxypurinol which has potentially adverse effects for patients. Considering these results, we propose the single-dose method currently used to treat hyperuricemia can result in unnecessarily high levels of allopurinol. While the short half-life of allopurinol in blood suggests that oxypurinol is responsible for enzyme inhibition, we anticipate multiple, smaller doses of allopurinol would reduce the total allopurinol patient load.

Allopurinol, an analog of hypoxanthine, has been used as an antigout drug for more than half a century and has been found to be generally effective in lowering uric acid levels in the blood (1). Allopurinol inhibits xanthine oxidoreductase (XOR), which catalyzes the final two steps of purine catabolism, the oxidative hydroxylation of hypoxanthine to xanthine and xanthine to uric acid. In mammalian tissues, including humans, XOR exists as either xanthine dehydrogenase (XDH) or xanthine oxidase (XO), two forms of the same gene product (2).

The most efficacious dosage of allopurinol, however, is not well-established. Even now, there are conflicting reports in the literature regarding dosage (3, 4, 5). Historically, allopurinol administration has been divided into two or three doses totaling 300 mg/day, although more recently a single daily dose of 300 mg has become more common (6, 7). However, many patients do not respond well to allopurinol and require doses as high as 800 to 900 mg/day. In recent clinical trials, single high doses of allopurinol rather than multiple lower doses have been used to facilitate comparison with febuxostat, a more recently introduced XOR inhibitor (8, 9). The assumption behind such a dosing regimen is apparently that oxypurinol, the principal metabolite of allopurinol, accumulation in the blood is the main effector of XOR inhibition, exerting an effect comparable to that of allopurinol.

Allopurinol is oxidatively hydroxylated to oxypurinol at the molybdenum center of XOR in the same manner as the usual purine hydroxylation reaction, resulting in the reduction of the active site molybdenum center from Mo(VI) to Mo(IV), as illustrated in Figure 1 (10, 11). Mo(IV) accumulates to only a small extent in the course of turnover because the electrons introduced into Mo(VI) are very rapidly transferred to the flavin adenine dinucleotide cofactor (*via* the two [2Fe-2S] clusters) and on to NAD^+^ (XDH) or O_2_ (XO), as shown in Figure 1.

**Figure 1:**
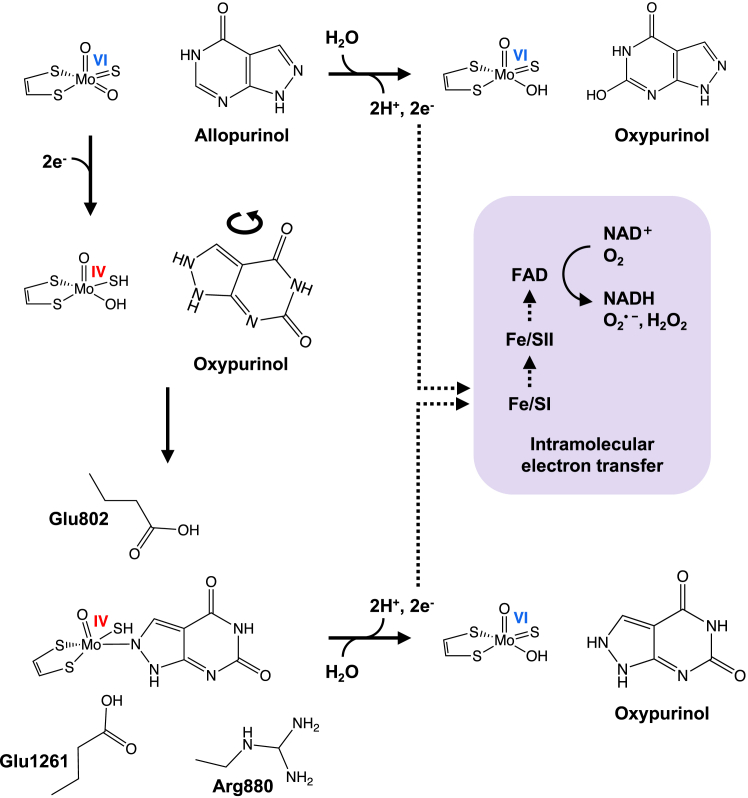
**The reported mechanism of inhibition of XOR by allopurinol.** Allopurinol is converted to oxypurinol at the molybdenum center of XOR, and oxygen is received from the water molecule (*top*). The electrons are very rapidly transferred to the FAD cofactor (*via* the two [2Fe-2S] clusters) and on to NAD^+^ (XDH) or O_2_ (XO). In the course of the reaction, molybdenum is reduced from Mo(VI) to Mo(IV) by taking an electron from the purine as a substrate, that is, allopurinol (*middle*). The Mo(IV) thus formed occurs only transiently, but when oxypurinol is properly oriented, it forms an enzyme-inhibitor complex (*bottom*). The reduced XOR (E_red_)•oxypurinol complex is gradually re-oxidized and oxypurinol dissociates. XO, xanthine oxidase; XOR, xanthine oxidoreductase.

Conversion of allopurinol to oxypurinol involves hydroxylation at N8 of the allopurinol inhibitor, leading to the coordination of oxypurinol to the molybdenum center *via* a nitrogen of the pyrazole subnucleus (12). Thus, oxypurinol, once generated, must rotate within the substrate binding site (Fig. 1) to form the inhibitory complex, but once properly oriented, it binds to the reduced molybdenum with great affinity. Allopurinol is thus a potent suicide inhibitor and its inhibition proceeds in a time-dependent manner (though in milliseconds) that can be observed by the stopped-flow method (10). The reduced XOR (E_red_)•oxypurinol complex is gradually reoxidized, at which point the oxypurinol dissociates; the half-life for this process is 300 min at pH 8.5 and 25 °C (10, 13). Oxypurinol thus produced and released into the blood has been assumed to reduce serum uric acid levels until it is cleared in the kidneys (14, 15, 16). Although the pharmacokinetics of oxypurinol have been reported (17), quantitative comparisons of the inhibitory powers and mechanisms of action of allopurinol *versus* oxypurinol have not been performed. Of particular interest is not only the extent of inhibition of the xanthine to uric acid reaction but also the conversion of hypoxanthine to xanthine, which is even more important for decreasing uric acid levels, as described further in the Discussion section.

In the present study, we compare the differential effectiveness of allopurinol and oxypurinol as inhibitors of XOR, administering each by direct intraperitoneal administration to mice so as to minimize the conversion of allopurinol to oxypurinol *via* the intestine and liver XOR. We also examine their respective mechanisms of inhibition through detailed kinetic and structural studies using highly active XO and XDH forms of the bovine enzyme.

## Results

### Effect of oxypurinol in a mouse model of hyperuricemia

**Figure 2:**
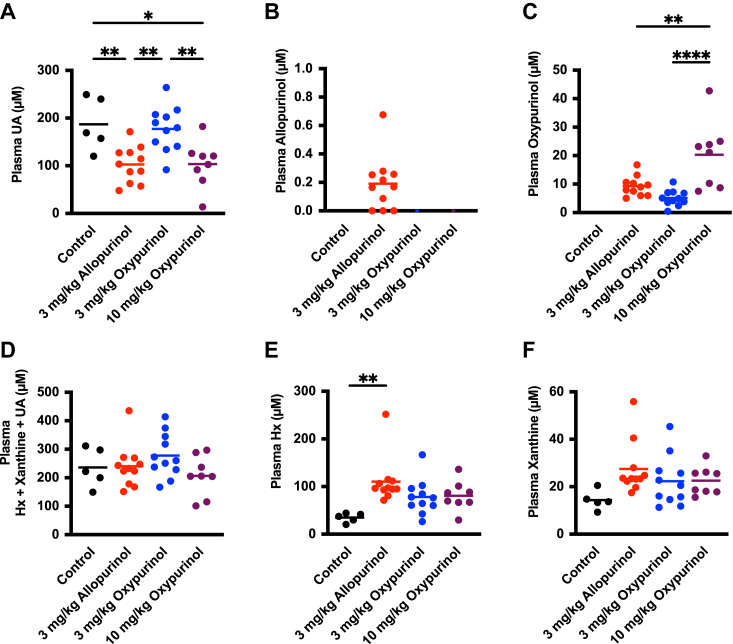
**Comparison of the effects of allopurinol and oxypurinol in a model of hyperuricemia.** Uric acid (*A*), allopurinol (*B*), oxypurinol (*C*), sum of hypoxanthine, xanthine, and uric acid (*D*), hypoxanthine (*E*), and xanthine (*F*) levels in plasma of mice treated with control (n = 5, *black*), 3 mg/kg allopurinol (n = 11, *red*), 3 mg/kg oxypurinol (n = 11, *blue*), 10 mg/kg oxypurinol (n = 8, *purple*). Statistical analysis was performed using one-way ANOVA and Turkey’s multiple comparison test. ∗*p* < 0.05, ∗∗*p* < 0.01, ∗∗∗∗*p* < 0.0001.

To compare the inhibitory effect of allopurinol and oxypurinol on uric acid production, mice were treated with oxonate to inhibit uricase and induce hyperuricemia. Treatment with 3 mg/kg of allopurinol, administered by peritoneal injection, significantly decreased plasma uric acid levels compared to the control group (Fig. 2*A*). One hour after administration, little allopurinol was detected in the plasma, but a small amount of oxypurinol was also observed (Fig. 2, *B* and *C*). In contrast, administration of 3 mg/kg of oxypurinol did not significantly reduce plasma uric acid levels compared to controls (Fig. 2*A*). Increasing the oxypurinol dose to 10 mg/kg was required to reduce plasma uric acid to that seen with 3 mg/kg allopurinol (Fig. 2*C*). The total plasma hypoxanthine, xanthine, and uric acid concentrations were similar (Fig. 2*D*). Allopurinol treatment significantly increased plasma hypoxanthine levels (Fig. 2*E*), although xanthine levels were largely unaffected (Fig. 2*F*). At both 3 and 10 mg/kg oxypurinol, neither hypoxanthine nor xanthine concentrations were significantly different from the control group.

### Comparison of the inhibitory effects of allopurinol and oxypurinol on uric acid production

**Figure 3:**
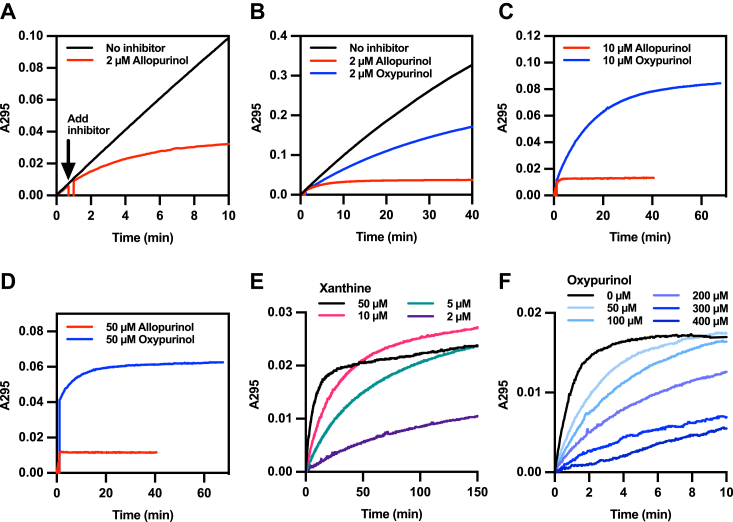
**Time-dependent inhibition.***A*–*D*, 2.38 nM XO type enzyme was added in 0.1 M pyrophosphate/acetic acid buffer pH 8.5, 0.2 mM EDTA, 50 μM xanthine, with 2 to 50 μM allopurinol or oxypurinol added in the course of the reaction at 25 °C. A295 indicates the concentration of uric acid yielded by XOR. *E*, the same condition as in (*A*–*D*) except that the concentrations used were 50 μM oxypurinol and 2 to 50 μM xanthine. Uric acid production was measured at 295 nm at 25 °C. *F*, the same enzyme was added to 0.1 M KPB (pH 7.4), 0.2 mM EDTA, and 2 μM xanthine, using 0 to 400 μM oxypurinol. KPB, potassium phosphate buffer; XO, xanthine oxidase; XOR, xanthine oxidoreductase.

The time dependence of uric acid production from xanthine using XOR *in vitro* under various conditions is shown in Figure 3. When XO reacts with 50 μM xanthine in the absence of inhibitors, uric acid production increases linearly at a rate of 90.3 μmol/μmol enzyme/min for about 40 min, as shown in Figure 3, *A* and *B*. After that, the rate of uric acid production gradually decreases as the substrate concentration decreases. The addition of 2 μM allopurinol 1 min after the start of the reaction inhibits uric acid production in a time-dependent manner (Fig. 3, *A* and *B*), and results in complete inhibition after 10 min. In addition, increasing the allopurinol concentration to 10 μM or 50 μM stops uric acid production immediately (Fig. 3, *C* and *D*). Oxypurinol also inhibits under these conditions, but it takes a significantly longer time and requires much higher concentrations to achieve the same level of inhibition. Similar results are obtained using both XO- and XDH-type enzymes (Fig. S1).

The results of these experiments are summarized in Table 1, which shows the ratio of time required for 50% inhibition (Table 1A) for allopurinol and oxypurinol and the ratio of time for complete inhibition of uric acid production (Table 1B) at the same concentration. Allopurinol is seen to be a more effective inhibitor by a factor of 10- to 30-fold for 50% inhibition and even more so for 100% inhibition. At high concentrations of allopurinol, the production of uric acid stops immediately under the assay conditions (noted as “NA” in Table 1). The time required for 50% inhibition by oxypurinol increases as the concentration of xanthine and oxypurinol increases, but this takes a very long time even at the higher concentrations. The time to completely inhibit uric acid production with oxypurinol could not be accurately measured (“Nd” in Table 1) within the instrument’s measurement time (9999 s). It is obvious from these results that allopurinol is the much more effective inhibitor.

**Table 1:** The inhibition of uric acid production

A. 50% inhibition time of uric acid production			
Substrate: Xanthine	Inhibitor: oxypurinol (min)/allopurinol (min) [ratio]		
	2 μM	10 μM	50 μM
2 μM	9.0/NA	14.9/NA	49.5/NA
5 μM	10.8/1.0 [10.8]	20.1/NA	38.3/NA
10 μM	15.3/1.4 [10.9]	20.8/NA	19.7/NA
50 μM	32.7/3.8 [8.6]	13.0/1.2 [10.8]	6.8/0.2 [34.0]

B. The stop time of uric acid production			
Substrate: Xanthine	Inhibitor: oxypurinol (min)/allopurinol (min) [ratio]		
	2 μM	10 μM	50 μM
2 μM	58.3/NA	89.0/NA	Nd/NA
5 μM	77.7/3.0 [25.9]	119.4/NA	Nd/NA
10 μM	101.4/6.9 [14.7]	Nd/NA	Nd/NA
50 μM	Nd/14.9	Nd/4.5	Nd/0.7

The time (minutes) was shown for each concentration of oxypurinol and allopurinol.

[]: The ratio value obtained.

NA: Uric acid production stopped instantaneously and could not be detected.

Nd: The curve did not completely level out within the measurement time.

### Enzyme kinetics of oxypurinol in the presence of xanthine substrate

The relationship between oxypurinol and xanthine concentration was examined further. As shown in Figure 3*E*, at constant oxypurinol concentration, higher xanthine concentrations accelerated the rate of uric acid formation, but the inhibitory effect increased over time and with a concomitant decrease in product formation at 50 μM *versus* 10 μM xanthine; the corresponding graphs cross each other after about 50 min. However, since the actual xanthine concentration in the body is known to be low (18), we examined the inhibitory effect of oxypurinol under physiological conditions with a constant concentration of 2 μM xanthine. At this low a xanthine concentration, uric acid production was evident even with 400 μM oxypurinol (Fig. 3*F*), and the mode of inhibition was strictly competitive in nature. These results indicate that allopurinol is more effective than oxypurinol, especially under physiological conditions.

Initial-velocity steady-state kinetics using xanthine as a substrate were performed with both XDH and XO (Table 2 and Fig. S2, *A* and *B*). A competitive pattern of inhibition was observed in both cases, and there was no significant difference in Ki between the XO and XDH forms of the enzyme. With both XO and XDH, the Ki for oxypurinol was about 10-fold higher than for allopurinol for both forms of the enzyme (19), indicating that XOR has a much higher affinity for allopurinol than for oxypurinol. This result is analogous to studies showing that hypoxanthine has a relatively higher enzyme affinity than xanthine, since allopurinol bears the same structural relationship to oxypurinol that hypoxanthine does to xanthine (20, 21).

**Table 2:** *K*_*i*_ values and types of inhibition

Enzyme	Substrate	Inhibitor	Electron acceptor	Detection wavelength	Types of inhibition	*K*_*i*_ (μM)
XO	Xanthine	Oxypurinol	O_2_	295 nm	Competitive	6.35 ± 0.96
XDH	Xanthine	Oxypurinol	NAD^+^	295 nm	Competitive	4.60 ± 0.87
XDH	Hypoxanthine	Oxypurinol	NAD^+^	340 nm	Competitive	3.15 ± 0.22
XOR	Xanthine	Oxypurinol	PMS	550 nm	Competitive	1.65 ± 0.24
XOR	Hypoxanthine	Oxypurinol	PMS	550 nm	Competitive	1.29 ± 0.14
XOR	Xanthine	Febuxostat	PMS	550 nm	Mixed	0.64 ± 0.12 (nM)
XOR	Hypoxanthine	Febuxostat	PMS	550 nm	Mixed	0.72 ± 0.19 (nM)

Values reported are the mean ± S.E.

### Inhibition of allopurinol and oxypurinol on the oxidation of hypoxanthine to xanthine

**Figure 4:**
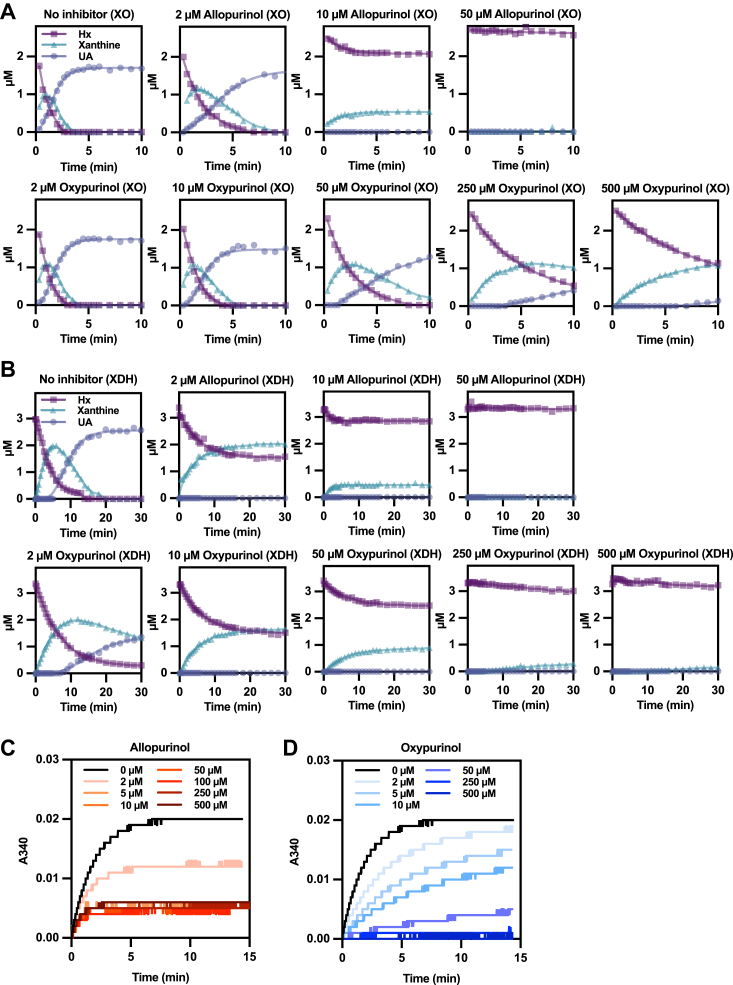
**XO and XDH kinetics of hypoxanthine substrate.***A*, the enzyme activity was assayed using 2 to 2.5 μM hypoxanthine as substrate in 0.1 M KPB pH 7.4, 0.2 mM EDTA, and 5 nM XO. No inhibitor, 2 to 50 μM allopurinol, and 2 to 500 μM oxypurinol were added to the reaction solution and reacted at 25 °C. Hypoxanthine, xanthine, and uric acid were measured by HPLC. *B*, the enzyme activity was assayed using 3 to 3.5 μM hypoxanthine as substrate in 0.1 M pyrophosphate/acetic acid buffer pH 8.5, 0.2 mM EDTA, and 3 nM XDH. No inhibitor, 2 to 50 μM allopurinol, and 2 to 500 μM oxypurinol were added to the reaction solution and reacted at 25 °C. Hypoxanthine, xanthine, and uric acid were measured by HPLC. *C* and *D*, the enzyme activity was assayed using 2 μM hypoxanthine as substrate in 0.1 M pyrophosphate/acetic acid buffer pH 8.5, 0.2 mM EDTA, 500 μM β-NAD^+^, and 6 nM XDH. 0 to 500 μM allopurinol (*C*) and 0 to 500 μM oxypurinol (*D*) were added to the reaction solution and reacted at 25 °C. The rate of NAD^+^ conversion to NADH was measured at A340 nm. KPB, potassium phosphate buffer; XDH, xanthine dehydrogenase; XO, xanthine oxidase.

We next investigated the effect of allopurinol and oxypurinol on the oxidation of hypoxanthine to xanthine by XOR. As shown in Figure 4, *A* and *B*, the reaction from hypoxanthine occurs stepwise, first to xanthine and then to uric acid. The addition of allopurinol at a concentration of 10 μM inhibited the conversion of 2.5 μM hypoxanthine to xanthine in about 5 min, and at 50 μM, inhibition was immediate, reflecting the formation of the tightly bound E_red_•oxypurinol complex upon oxidation of the allopurinol (Fig. 4*A*). While oxypurinol delayed the formation of uric acid the accumulation of xanthine was still significant even at 500 μM oxypurinol. A similar trend was observed with both XO and XDH forms of the enzyme (Fig. 4*B*).

The inhibitory effect of allopurinol and oxypurinol on the reduction of NAD^+^ to NADH by XDH is shown in Figure 4, *C* and *D*. In this experiment, the two-step reaction of hypoxanthine to uric acid, one equivalent of NADH is generated with each step. Above 5 μM, allopurinol immediately inhibited NADH formation, reflecting the formation of the tightly bound E_red_•oxypurinol complex upon oxidation of the allopurinol (Fig. 4*C*). In contrast, inhibition by oxypurinol required higher concentrations to achieve the same inhibitory effect as allopurinol (Fig. 4*D*). As shown in Table 2 and Fig. S2*C*, when hypoxanthine was used as a substrate, oxypurinol showed the same mode of inhibition as with xanthine as substrate, and Ki values were also similar.

Initial velocity analysis using oxygen or NAD^+^ as the electron acceptor includes the effect of time-dependent coordination bond formation. Therefore, phenazine methosulfate was used to induce direct, rapid reoxidation of the molybdenum center (22, 23). Under these artificial conditions, oxypurinol exhibited a competitive mode of inhibition against the hypoxanthine substrate, as shown in Fig. S2*E*, with a Ki of 1.29 μM (Table 2).

### The affinity of enzyme for substrate

The *K*_*m*_ values of each substrate were measured and are shown in Table 3. The *K*_*m*_ of XO for xanthine was similar to that found in previous studies (21, 24). The *K*_m_ of XDH for xanthine was lower than that of XO, and the *K*_*m*_ for hypoxanthine lower still. This result can be explained by the fact that hypoxanthine is the better substrate for XOR by both spectrophotometric (20, 21, 25) and HPLC (26) analyses. In the case of allopurinol, *K*_*m*_ was between hypoxanthine ad xanthine, but *k*_cat_/*K*_*m*_ was lower, suggesting that it was affected by the suicide substrate. Therefore, we performed an enzymatic reaction with phenazine methosulfate and obtained the same parameters, which showed that allopurinol is a good substrate equivalent to hypoxanthine.

**Table 3:** Kinetic parameters for the reaction of XOR from bovine milk

Enzyme	Substrate	Electron acceptor	Detection wavelength	*K*_*m*_ (μM)	*k*_cat_ (s^−1^)	*k*_cat_/*K*_*m*_ (s^−1^/μM)
XO	Xanthine	O_2_	295 nm	11.51 ± 1.18	14.06 ± 0.41	1.22
XDH	Xanthine	NAD^+^	340 nm	5.29 ± 0.48	11.08 ± 0.38	2.10
XDH	Hypoxanthine	NAD^+^	340 nm	2.64 ± 0.29	6.37 ± 0.28	2.42
XDH	Allopurinol	NAD^+^	340 nm	4.23 ± 0.52	1.83 ± 0.92	0.43
XOR	Xanthine	PMS	550 nm	24.02 ± 2.49	40.78 ± 1.33	1.70
XOR	Hypoxanthine	PMS	550 nm	5.29 ± 0.24	34.20 ± 0.39	6.47
XOR	Allopurinol	PMS	550 nm	3.59 ± 0.45	26.17 ± 1.96	7.30

Values reported are the mean ± S.E.

### The half-life of the E_red_•oxypurinol complex

**Figure 5:**
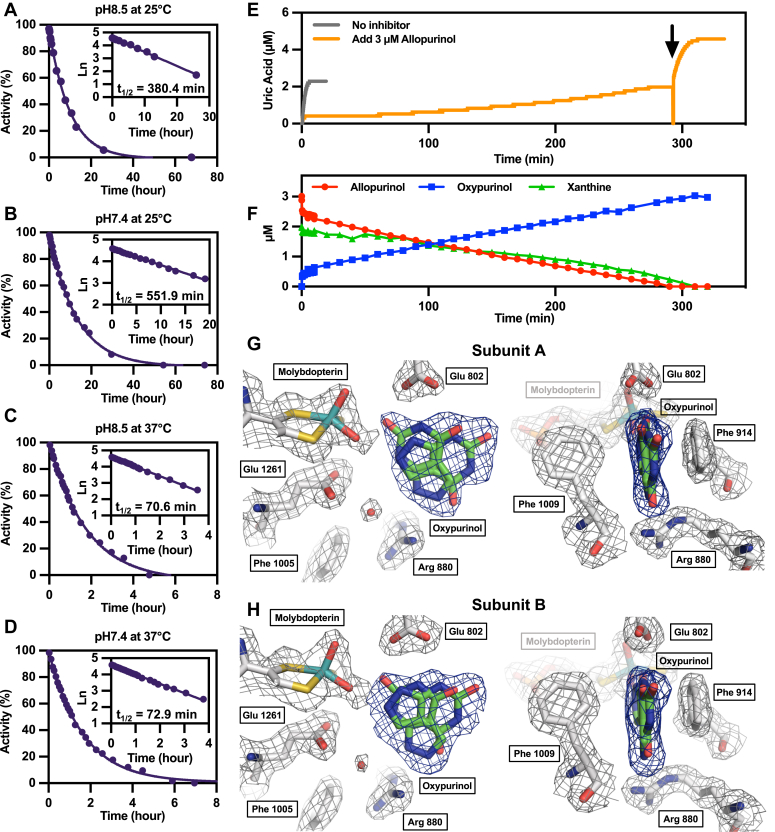
**Half-life of the complex of XOR-oxypurinol and crystal structure.***A*–*D,* Oxypurinol-complexed XO type enzyme was incubated at 25 °C and 37 °C, respectively. Enzyme incubated at each temperature was added to 0.1 M pyrophosphate/acetic acid buffer pH 8.5, 0.2 mM EDTA, or 0.1 M KPB pH 7.4, 0.2 mM EDTA, both buffers containing 150 μM xanthine. Uric acid production was measured at 295 nm at 25 °C. The activity recovery was followed overtime until the AFR reached approximately 200. The main figure plots activity (%) against time. Using this plot as a primary reaction, Ln was plotted against time (*inset*), and the half-life was determined from the slope of the straight line. *E*, an enzyme reaction solution consisting of 0.1 M KPB pH 7.4, 0.2 mM EDTA, 2.56 nM XO, 2 μM xanthine, 3 μM allopurinol was incubated at 37 °C, and uric acid production was followed by 295 nm. Two micromolar xanthine was added at the time indicated by the *arrow*. *F*, HPLC measured allopurinol, oxypurinol, and xanthine under the same reaction conditions with *E*. *G* and *H*, crystal structure of the active sites in subunits A and B of the E_ox_•oxypurinol complex. The two orientations of the oxypurinol molecules that best explain the corresponding polder electron density maps (*blue*; 5 σ level) are shown, in (*G*) subunit A [occupancies of 0.58 and 0.42 for orientations 1 and 2, respectively] and (*H*) subunit B [occupancies of 0.56 and 0.44 for orientations 1 and 2, respectively]. Neighboring side chains and the molybdopterin cofactor together with their 2F_o_-F_c_ electron density (*gray*; 1.0 σ level) are also displayed. AFR, activity to flavin ratio; KPB, potassium phosphate buffer; XO, xanthine oxidase; XOR, xanthine oxidoreductase.

We next examined the half-life of the E_red_•oxypurinol complex under more physiological conditions. Consistent with previously reported results (10, 13), the half-life was approximately 380 min at pH 8.5 and 25 °C (Fig. 5*A*), increasing to 552 min at pH 7.4 and 25 °C (Fig. 5*B*); the half-life at both pH values was shortened to 70 min at 37 °C (Fig. 5, *C* and *D*).

In another experiment, XO was added to a reaction mix containing both allopurinol and xanthine, monitoring the reaction both spectrophotometrically and by HPLC over an extended period (Fig. 5, *E* and *F*). In the absence of inhibitors, 2 μM xanthine was completely converted to uric acid in 6 min (Fig. 5*E*). The addition of 3 μM allopurinol resulted in the immediate accumulation of 0.5 μM oxypurinol (Fig. 5*F*), with formation of the E_red_•oxypurinol stopping the conversion of xanthine to uric acid (Fig. 5*E*). Uric acid production was stopped for up to 60 min, during which time allopurinol continued to react (Fig. 5*F*). Xanthine was completely converted to uric acid at 300 min, and the subsequent addition of 2 μM xanthine resulted in a plot similar to that with no inhibitor (Fig. 5*E*). Thus, free oxypurinol, once dissociated from the enzyme contributed little to inhibition. Further addition of allopurinol was necessary to maintain inhibition of uric acid formation.

### The mode of oxypurinol binding to oxidized XOR

The crystal structure of the oxidized XOR (E_ox_) in complex with oxypurinol has been determined (Table 4). Unlike the dative bond seen with Mo(IV) in E_red_ (Fig. 1), oxypurinol does not coordinate directly to Mo(VI) of E_ox_, but still sits in the substrate binding site. The overall location of the aromatic substrate system is quite clear; sandwiched between the two phenyl rings of Phe914 (parallel) and Phe1009 (edge-on) but, based on the electron density, appears able to assume two or three discrete orientations (rotated around an axis perpendicular to the center of the aromatic ring system). In Figure 5, *G* and *H*, two orientations are shown that performed well in the crystallographic refinement procedure. All possible oxypurinol orientations interfere with substrate access to the active site. This behavior is consistent with XOR's very broad substrate specificity and its ability to bind quite a variety of compounds (27). The nature of the oxypurinol binding mode is typical of that of many weak inhibitors of XOR and contrasts with the clear binding modes seen with tightly bound inhibitors (12, 22, 23, 28, 29) or substrate reaction intermediates (30).

**Table 4:** X-ray crystallographic data collection and refinement statistics

Data collection	
PDB entry	8J79
Beamline	SPring-8 BL44XU
Wavelength (Å)	0.90000
Resolution range (Å)	48.43–1.99 (2.11–1.99)
Space group	*C*2
Unit cell parameters (Å, °)	*a* = 167.68, *b* = 123.47, *c* = 150.06, *β* = 90.9
Total reflections	1,049,285 (166,138)
Unique reflections	210,046 (33,646)
Multiplicity	5.0 (4.9)
Data completeness (%)	99.7 (99.1)
*I/σ(I)*	10.6 (2.6)
*R*_merge_	0.097 (0.54)
CC_1/2_	0.996 (0.843)
Refinement	
Resolution range (Å)	47.72–1.99 (2.04–1.99)
Reflections, refinement	199,435 (14,500)
Reflections, *R*_free_	10,629
*R*_work_	0.163 (0.241)
*R*_free_	0.197 (0.279)
Protein residues	2573 (2 chains)
Water molecules	1815
RMS (bonds) (Å)	0.01
RMS (angles) (°)	0.87
Ramachandran favored (%)	95.4
Ramachandran allowed (%)	3.8
Ramachandran disallowed (%)	0.8
Rotamer outliers (%)	0.9
Average *B*-factor (Å^2^) overall	32.3
Protein	31.7
Ligands	31.9
Water	38.5

Values in parentheses are for the outermost shells.

### Effect of oxypurinol on purine nucleoside phosphorylase

Allopurinol also is a substrate of hypoxanthine-guanine phosphoribosyltransferase (HGPRT) (31), but oxypurinol was neither a substrate nor an inhibitor of HGPRT. Allopurinol and its ribosyl derivative are relatively weak inhibitors of purine nucleoside phosphorylase (PNP) with *K*_*i*_ >200 μM (32, 33). Plasma allopurinol never reaches that concentration, but oxypurinol concentrations could (34, 35). Although oxypurinol has been reported to have a stronger inhibitory effect than allopurinol (32, 36), the results were obtained prior to several reports on the cooperativity of PNP (37). Fig. S3*A* shows the inosine degradation reaction by PNP as a function of reaction rate and substrate concentration; the Lineweaver-Burk plot curves with increasing inosine concentration, consistent with previous reports (38). Although oxypurinol exhibits a competitive mode of inhibition for inosine, a plot of 1/v *versus* [oxypurinol] was nonlinear (Fig. S3, *B* and *C*). This nonideal behavior may be a manifestation of the well-established allosteric properties of PNP (37).

## Discussion

**Figure 6:**
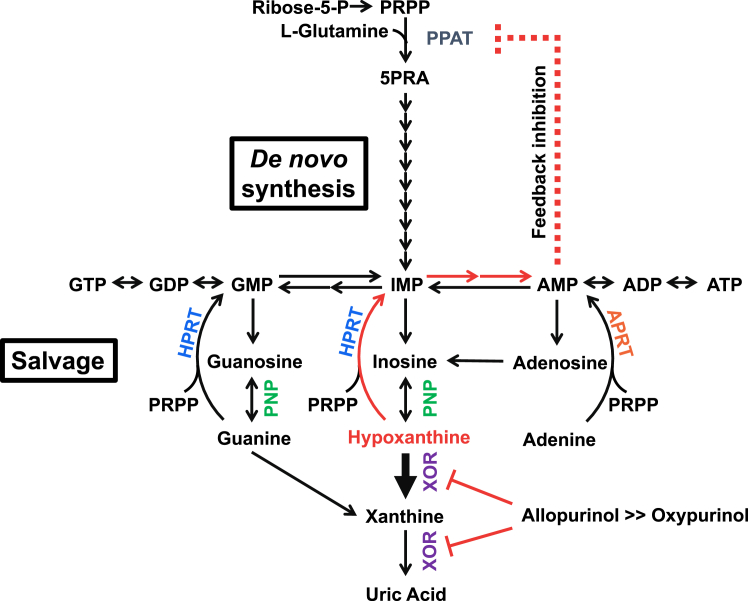
**Purine metabolism**.

Since the discovery of allopurinol, several inhibitors of XOR have been developed (39). Some of these agents (*e.g.*, BOF and Y700) have a *K*_*i*_ lower than 10^−8^ M for the conversion of xanthine to uric acid (22, 28), but do not exhibit a clinical efficacy comparable to allopurinol. Steady-state kinetic analysis shows that allopurinol has a *K*_*i*_ of 10^−7^ M for the xanthine to uric acid reaction, but its true mode of action is that of a suicide substrate: once oxidized to oxypurinol, it reorients in the enzyme active site and binds tightly to the still-reduced molybdenum center. The importance of its inhibitory mechanism may lie in the strong inhibition of the conversion of hypoxanthine to xanthine (Fig. 6). The two most clinically useful inhibitors developed in recent years, febuxostat and topiroxostat, both have sub-nM *K*_*d*_ and *K*_*m*_ values for inhibition of hypoxanthine oxidation (Fig. S4) (23, 29). As an example of the complete inhibition of XOR, consider the metabolism in the case of XOR deficiency, in which the patient has significantly reduced serum uric acid levels and urinary uric acid excretion accompanied by increased urinary xanthine levels (40). This results in xanthinuria and xanthine stones but no other severe symptoms (41, 42, 43). Plasma hypoxanthine levels, on the other hand, are not significantly increased, due to salvage by HGPRT (Fig. 6) (40). Salvaged AMP (*via* IMP) inhibits phosphoribosyl pyrophosphate amidotransferase, the rate-limiting enzyme in the *de novo* purine biosynthesis pathway, by an allosteric feedback mechanism (44, 45). That is, it suppresses purine nucleotide synthesis. Thus, inhibition of hypoxanthine conversion to xanthine is essential for effective lowering of uric acid production. The results of either allopurinol or oxypurinol administration in the hyperuricemic mouse model in this study were obtained 1 h after administration and considered to be solely due to the inhibition of XOR. Longer-term experiments would be desirable to validate uric acid reduction by feedback inhibition, but renal toxicity due to xanthine deposition was a limiting factor (46). Purine metabolism differs among species, and high HGPRT activity–uricase KO mice have been developed to study human purine metabolism (47). Humans with high HGPRT activity are expected to exhibit more uric acid-lowering effects.

The mechanism by which oxypurinol inhibits XOR is different from that of allopurinol. Oxypurinol alone cannot reduce XOR and therefore does not form a particularly tight inhibition complex as confirmed by the results of our present structural analysis. In the presence of hypoxanthine and xanthine, however, the enzyme becomes reduced and the formation of a tight-binding complex is possible. Hypoxanthine has a high affinity for the enzyme and is readily hydroxylated to xanthine by XOR. The resulting xanthine is not immediately hydroxylated to uric acid, but must dissociate from the enzyme before rebinding to the enzyme for oxidation to uric acid. Therefore, in order to encounter a reduced molybdenum center, oxypurinol must enter the binding pocket after the substrate dissociates, but because of its low affinity a high concentration is required to do so and thereby inhibit hydroxylation. On the other hand, allopurinol has a *K*_*m*_ and *k*_cat_/*K*_*m*_ similar to that of hypoxanthine and is an excellent substrate.

For oxypurinol to bind tightly, the molybdenum must be reduced. However, *in vivo* levels of hypoxanthine and xanthine are usually low and vary with ATP consumption, exercise, and diet (48, 49, 50). XOR is thus expected to be largely oxidized, although this may vary from patient to patient resulting in different levels of inhibition. High oxypurinol concentrations do lower uric acid production, but the inhibitory effect on XOR is merely competitive. This suggests that high concentrations of oxypurinol may in fact delay the formation of the tightly bound inhibitory complex with the reduced form of the enzyme, most probably by blocking access of the substrate to the active site and thereby inhibiting the reduction of the enzyme. Given that oxypurinol contributes to reducing uric acid production through competitive inhibition, xanthine rather than hypoxanthine should accumulate. In this case, purine synthesis is not inhibited because no feedback inhibition of the *de novo* pathway occurs. High xanthine accumulation could increase the risk of xanthinuria and xanthine stone formation when oxypurinol is used to treat hyperuricemia (41). On the other hand, such adverse effects have not been reported with allopurinol in patients with no history of hereditary disease such as HGPRT deficiency which enhances the *de novo* synthesis and results in hyperuricemia (51).

Allopurinol exerts its inhibitory effect primarily in the liver, where XOR is expressed, but is also converted to oxypurinol by aldehyde oxidase without inhibition of the enzyme (52, 53). Allopurinol is rapidly metabolized in the liver and rarely accumulates in the blood (54). Since XOR is not normally present there, any XOR activity found is most likely due to the enzyme released from other tissues and oxypurinol accumulated in the blood is unlikely to contribute to inhibition.

In managing gout, the dose of allopurinol needed to achieve target levels of serum uric acid varies from individual to individual (55). Current guidelines recommend lowering serum uric acid levels to less than 6 mg/dl (0.36 mmol/l) (56). However, many patients with gout have difficulty achieving such levels of serum uric acid with allopurinol dosages based on renal function. Since allopurinol is a well-tolerated and relatively safe drug, doses can be increased in refractory patients (55), although some patients are at increased risk of severe cutaneous adverse drug reactions such as Stevens-Johnson syndrome/toxic epidermal necrolysis (57). It has been suggested that the possession of the HLA-B∗58:01 allele and T cell-mediated immune responses are involved in the pathogenesis of adverse effects observed with oxypurinol (58, 59). The present study confirms that oxypurinol weakly but allosterically inhibits PNP, which is expressed at high levels in lymphoid tissues (60). PNP deficiency has been shown to induce immunodeficiency, principally through T cell dysfunction (36, 60, 61). This association necessitates further investigation of the immune response mediated by oxypurinol *via* PNP.

Attempts have been made to assess allopurinol dosage by measuring oxypurinol in plasma (34, 62) and identify the upper limit of the therapeutic range for oxypurinol (63). Due to the complexity of the mechanism of action, however, some misunderstanding seems to have arisen. Graham *et al.* (64) report that the oxypurinol concentration at which xanthine oxidase is maximally inhibited is 33.75 μM. Therapeutic serum oxypurinol concentrations are 30 to 100 μmol/l (34, 62). This is considered an acceptable concentration of oxypurinol derived from allopurinol, which is involved in complex formation. Recently, Stamp *et al.* found that higher oxypurinol concentrations (>100 μmol/l) are necessary to achieve target serum uric acid levels and that it is safer to increase allopurinol dosage rather than dosage based on renal function even in people with chronic kidney disease (5, 16). However, this view is questionable given the mechanism of inhibition, and it is unclear whether maintenance of blood oxypurinol levels at that concentration is in fact sufficient to lower serum uric acid levels.

Excess allopurinol is present in the blood in high-dose single-dose regimens, and tissue XOR may be fully inhibited under these conditions, and the inhibitor repeatedly binds to and dissociates from the active sited under these conditions. Although uric acid production has apparently ceased, allopurinol continues to be almost completely metabolized to oxypurinol because of its effectiveness as a substrate. Allopurinol levels are thus expected to continue to increase despite having already achieved therapeutic serum oxypurinol levels (30–100 μmol/l) and effective inhibition of uric acid production (34, 62). It should be noted that the uric acid-lowering effect of allopurinol is due to its suicide inhibition of XOR, and not to the accumulation of free oxypurinol. This being the case, while monitoring plasma oxypurinol concentrations can effectively assess patient adherence to medical allopurinol dosage it is not itself sufficient to assess the efficacy of oxypurinol in lowering serum uric acid. Oxypurinol is not the sole inhibitor accumulating in blood based on statistical calculations as allopurinol is itself present (15). This is a particularly important consideration in the liver where XOR is present in high levels and where the per os administered inhibitor should work first, as was previously reported in an allopurinol loading test (53).

The major clearance pathway for oxypurinol is through the kidneys, and patients with renal insufficiency are more likely to accumulate oxypurinol in the blood because their serum half-life of oxypurinol is significantly prolonged. Patients receiving 300 mg allopurinol daily for more than 2 weeks had serum oxypurinol concentrations greater than 100 μM (34), which is above the critical value for PNP inhibition, as determined in this study. In addition, with no clear guidelines for dose adjustment, we support the opinion of Laville *et al.* (3, 4) to limit allopurinol dosage based on renal function so as to avoid the prescription of excessive doses of allopurinol, described as allopurinol abuse (65). However, there is concern that the target level of serum uric acid may not be achieved. Our results indicate that constant readministration of small quantities of allopurinol is the more effective therapeutic regimen to maintain effective inhibition of both hypoxanthine and xanthine oxidation. Such split-dosing, considering the half-life of the enzyme and inhibitor, is expected to more effectively reduce the total amount of allopurinol needed to reduce serum uric acid levels while at the same time minimizing the accumulation of oxypurinol in the blood, thereby reducing the risk of long-lasting chronic conditions. In addition, smaller, more frequent dosing reduces the impact of a single missed dose, although there is some concern about the impact on cardiac mortality if doses are missed continuously (8).

Therefore, it is important to understand the inhibitory mechanism and its effect on purine metabolism when using XOR inhibitors. Even after the exact mechanisms of action of each drug for gout therapy are known, treatment choice will still have to consider an individual's life and personal characteristics, emphasizing the need for personalized medicine.

## Experimental procedures

### Effect of oxypurinol in hyperuricemia model

Six-week-old specific-pathogen free-grade male Institute of Cancer Research mice were purchased from Japan SLC. All animal experiments were performed according to the protocol approved by the Animal Care and Ethics Committee of Tokyo University of Pharmacy and Life Sciences (Permit No. P21-80). Mice were housed under conditions of 22 to 24 °C, 40 to 60% humidity, and a 12-h light/12-h dark cycle. Food and water were administered *ad libitum*.

Oxypurinol or allopurinol was suspended in 0.5% carboxymethyl cellulose and administered intraperitoneally at 3 mg/kg or 10 mg/kg (n = 11). The control group received 0.5% carboxymethyl cellulose (n = 5). Immediately after intraperitoneal administration, potassium oxonate was injected subcutaneously at 300 mg/kg. One hour after administration, mice were sacrificed by cervical dislocation, and blood was collected with heparin. Hypoxanthine, xanthine, uric acid, allopurinol, and oxypurinol in plasma were measured by HPLC. Statistical analysis was performed using GraphPad Prism 9 software (www.graphpad.com). Two mice treated with 10 mg/kg oxypurinol were excluded because no increased plasma oxypurinol was observed.

### Purification and enzyme assays of XOR

Bovine milk was obtained from the animal farm of the Nippon Veterinary and Life Science University. Highly active bovine XOR in the oxidase form (XO) was purified using affinity chromatography (66). The dehydrogenase form of the enzyme (XDH) was obtained by reacting XO immediately upon purification with 5 mM DTT at pH 8.5, 25 °C for 1 h. XO and XDH assays were carried out at 25 °C under aerobic conditions. The reaction solution was composed of 0.1 M pyrophosphate/acetic acid buffer (pH 8.5), 0.2 mM EDTA, various concentrations of xanthine, hypoxanthine, and inhibitor (allopurinol, oxypurinol, and febuxostat) in a final volume of 3 ml. A total of 500 μM β-NAD^+^ was added to the same reaction solution for XDH activity. Physiological conditions such as pH 7.4 (0.1 M potassium phosphate buffer (KPB), 0.2 mM EDTA) and the addition of 50 μM β-NADH were also used. The activity was measured by following 295 nm and 340 nm on a UV-1800 or UV1900i spectrophotometer. Kinetics in the reduction of cytochrome c by phenazine methosulfate binding was performed, using 0.1 M pyrophosphate/acetic acid buffer (pH 8.5), 0.2 mM EDTA, 20 μM phenazine methosulfate, 20 μM cytochrome c from bovine heart, 2 nM XOR, and various concentrations of hypoxanthine and oxypurinol by following changes in absorbance at 550 nm with a UV-1900i Spectrophotometer at 25 °C. In addition, the enzyme reaction solution was quenched with perchloric acid and neutralized with K_2_CO_3_, with products analyzed using HPLC as described below. Spectrophotometric data were analyzed using UV-Probe software Ver. 2.70 or LabSolutions UV-Vis Ver. 1.11 (Shimadzu). Kinetic parameters were determined from nonlinear curve fits of raw data using GraphPad Prism 9 software.

### The half-life of E_red_•oxypurinol

XO-type enzyme complexed with oxypurinol was incubated at 25 °C and 37 °C, in both 0.1 M pyrophosphate/acetic acid buffer pH 8.5, 0.2 mM EDTA or 0.1 M KPB pH 7.4, 0.2 mM EDTA; both assays contained 150 μM xanthine. Uric acid production was measured at 295 nm with UV-1800 Spectrophotometer at 25 °C. The activity recovery was followed overtime until the activity to flavin ratio reached approximately 200, indicating full reactivation.

### Crystallization, X-ray data collection, and refinement

Highly active XOR was incubated with 1 mM salicylate and 50 μM ferricyanide for 5 min at 25 °C in the dark. The enzyme solution was buffer exchanged into 50 mM Tris–HCl pH 7.4 and diluted with 50 mM Tris–HCl pH 7.4, 5 mM DTT, 100 μM oxypurinol, and 30% glycerol to a concentration of 8.6 mg/ml. The precipitant solution contained 50 mM potassium phosphate pH 6.0, 5 mM DTT, 0.2 mM EDTA, 100 μM oxypurinol, 30% glycerol and 14% to 15% PEG 4000. Ten microliters of enzyme solution and 10 μl of reservoir solution were mixed on siliconized glass plates and kept in the dark at 20 °C for 5 days. Diffraction data were collected at 100K on the BL44XU beamline (Spring-8) using 0.9 Å wavelength radiation and reduced with the X-ray detector software package (67). The crystal structure was determined by molecular replacement using the program MOLREP (68). The atomic models were built with COOT (69) and refined with CCP v. 6.1 (70) and phenix (71). Refinement did not include models for the ligand; electron density covering the oxypurinol molecule in Figure 5, *G* and *H* represents a polder map (72). Figures of the model were generated with PyMOL Molecular Graphics System (pymol.org/2), Version 2.0 Schrödinger, LLC. Statistics for data collection and structural refinement are shown in Table 4.

### PNP activity assay

Purine nucleoside phosphorylase (human, recombinantly expressed in *E. coli*) was purchased from Sigma-Aldrich. The reaction solution for inosine hydrolysis consisted of 50 mM KPB (pH 7.4), with varying concentrations of inosine and oxypurinol. The reaction was quenched with perchloric acid and neutralized by K_2_CO_3_, with hypoxanthine as products analyzed by HPLC.

### HPLC conditions

Purine concentrations were measured using a Shimadzu HPLC system consisting of the CBM-20A controller, LC-20AD pump, SIL-20AC autosampler (set at 4 °C), CTO-20A column oven (set at 40 °C), and SPD-M20A diode array detector. Peak analysis was performed using LC solution software. A SUPELCOSIL LC-18-T column (25 cm × 4.6 mm id 5 μm particle size) was used with the SUPELCOSIL LC-18-T Supelguard guard column separation of purines, using a flow rate was 0.8 ml/min and 10 or 20 μl samples. The mobile phase used was A: 0.1 M KPB and 4 mM tetrabutylammonium hydrogen sulfate (pH 5.5), B: 70% buffer A and 30% methanol (v/v). Gradient conditions were 0 min (0% B), 2 min (0% B), 4 min (30% B), 12 min (60% B), 15 min (100% B), 19 min (100% B), 20 min (0% B), and 30 min (0% B). Detection wavelengths were 254 nm, 268 nm, and 295 nm. The data were analyzed using LabSolutions Ver. 5.97 SP1. All purines were identified by retention time and quantified using an external standard.

## Data availability

All data are contained within the article. The crystal structure has been deposited in the Protein Data Bank under the accession code 8J79.

## Supporting information

This article contains supporting information.

## Conflict of interest

Takeshi Nishino is employed at NeSA LLC, a pharmaceutical consultancy company.

## Supporting information

Figure S1 **Time dependence of allopurinol and oxypurinol on XO and XDH.***A* and *B*, 2.38 nM XO type enzyme was added in 0.1 M pyrophosphate/acetic acid buffer pH 8.5, 0.2 mM EDTA, 50 μM xanthine, with 2 to 50 μM allopurinol (*A*) or oxypurinol (*B*) added in the course of the reaction at 25 °C. A295 indicates the concentration of uric acid yielded by XOR. *C* and *D*, 1.23 nM XDH type enzyme was added in 0.1 M pyrophosphate/acetic acid buffer pH 8.5, 0.2 mM EDTA, 50 μM xanthine, 50 μM NADH, 500 μM NAD^+^ with 2 to 50 μM allopurinol (*C*) or oxypurinol (*D*) added in the course of the reaction at 25 °C. A295 indicates the concentration of uric acid yielded by XOR. **Figure S2**. **The XOR inhibition mode of oxypurinol.***A*–*G*, Lineweaver-Burk plots were generated to visualize data and estimated the inhibition patterns. Data are representative of least two independent experiments. *A*, competitive inhibition of XO by oxypurinol using xanthine as substrate. The reactions were followed at 295 nm in 3 ml of solution containing various concentrations of xanthine, 0.1 M pyrophosphate/acetic acid buffer pH 8.5, 0.2 mM EDTA, and 2.38 nM XO in the absence or presence of oxypurinol. *B*, competitive inhibition of XDH by oxypurinol using as xanthine as substrate. The same as in *A* except that 1.23 nM XDH was used instead of XO and 500 μM NAD^+^ was added to the reaction solution. *C*, competitive inhibition of XDH by oxypurinol using hypoxanthine as substrate. The reation were followed at 340 nm in 3 ml of solution containing various concentration of hypoxanthine, 0.1 M pyrophosphate/acetic acid buffer pH 8.5, 0.2 mM EDTA, 500 μM NAD^+^ and 34 nM XDH in the absence or presence of oxypurinol. *D*, competitive inhibition of XOR by oxypurinol using xanthine as substrate. The reaction was followed at 550 nm in 3 ml of solution containing various concentration of xanthine, 0.1 M pyrophosphate/acetic acid buffer pH 8.5, 0.2 mM EDTA, 20 μM cytochrome c, 20 μM phenazine methosulfate and 2 nM XOR in the absence or presence of oxypurinol. *E*, competitive inhibition of XOR by oxypurinol using hypoxanthine as substrate. The same as in *D* except that hypoxanthine was used instead of xanthine. *F*, mixed-type inhibition of XOR by febuxostat using xanthine as substrate. The same as in *D* except that febuxostat was used instead of oxypurinol. *G*, mixed-type inhibition of XOR by febuxostat using hypoxanthine as substrate. The same as in *F* except that hypoxanthine was used instead of xanthine. **Figure S3**. **Allosteric inhibition of PNP by oxypurinol.***A*, kinetics of inosine decomposition reaction. Lineweaver-Burk plot and Michaelis-Menten plot (*inset*) to illustrate the cooperative model at the higher inosine concentrations (*red dashed line*). *B*, mode of inhibition of oxypurinol on the inosine degradation reaction of PNP. Lineweaver-Burk plots using 0, 350, 750 and 1000 μM oxypurinol as inhibitors showed a competitive mode. *C*, secondary plots obtained from Lineweaver-Burk indicating nonlinearities. 0, 250, 375, 500, 750 and 1000 μM of oxypurinol were used. **Figure S4**. **Inhibitory effects of febuxostat on hypoxanthine hydroxylation.***A*, the degradation of 15 μM hypoxanthine was followed overtime in 0.1 M KPB pH 7.4, 0.2 mM EDTA at 25 °C using 5 nM XO. To the reaction solution of the same composition, 1 to 100 nM febuxostat was added. Hypoxanthine, xanthine, and uric acid were measured by HPLC. *B*, degradation of 6 μM hypoxanthine was followed overtime at 25 °C in 0.1 M pyrophosphate/acetic acid buffer pH 8.5, 0.2 mM EDTA, 500 μM NAD^+^ using 3.4 nM XDH. Reactions of the same composition with 1 and 10 nM febuxostat were measured as in (*A*). *C*, the enzyme activity was assayed using 2 μM hypoxanthine as substrate in 0.1 M pyrophosphate/acetic acid buffer pH 8.5, 0.2 mM EDTA, 500 μM β-NAD^+^ and 6 nM XDH. 0 to 1 μM febuxostat were added to the reaction solution and reacted at 25 °C. The rate of NAD^+^ conversion to NADH was measured at A340 nm.
