# Supplementary material for: Allopurinol and oxypurinol differ in their strength and mechanisms of inhibition of xanthine oxidoreductase
Source: J Biol Chem. 2023 Aug 23;299(9):105189. doi: 10.1016/j.jbc.2023.105189 (PMC10511816; doi:10.1016/j.jbc.2023.105189)
Supplement: Figure S1 — Time dependence of allopurinol and oxypurinol on XO and XDH.A and B, 2.38 nM XO type enzyme was added in 0.1 M pyrophosphate/acetic acid buffer pH 8.5, 0.2 mM EDTA, 50 μM xanthine, with 2 to 50 μM allopurinol (A) or oxypurinol (B) added in the course of the reaction at 25 °C. A295 indicates the concentration of uric acid yielded by XOR. C and D, 1.23 nM XDH type enzyme was added in 0.1 M pyrophosphate/acetic acid buffer pH 8.5, 0.2 mM EDTA, 50 μM xanthine, 50 μM NADH, 500 μM NAD+ with 2 to 50 μM allopurinol (C) or oxypurinol (D) added in the course of the reaction at 25 °C. A295 indicates the concentration of uric acid yielded by XOR. Figure S2. The XOR inhibition mode of oxypurinol.A–G, Lineweaver-Burk plots were generated to visualize data and estimated the inhibition patterns. Data are representative of least two independent experiments. A, competitive inhibition of XO by oxypurinol using xanthine as substrate. The reactions were followed at 295 nm in 3 ml of solution containing various concentrations of xanthine, 0.1 M pyrophosphate/acetic acid buffer pH 8.5, 0.2 mM EDTA, and 2.38 nM XO in the absence or presence of oxypurinol. B, competitive inhibition of XDH by oxypurinol using as xanthine as substrate. The same as in A except that 1.23 nM XDH was used instead of XO and 500 μM NAD+ was added to the reaction solution. C, competitive inhibition of XDH by oxypurinol using hypoxanthine as substrate. The reation were followed at 340 nm in 3 ml of solution containing various concentration of hypoxanthine, 0.1 M pyrophosphate/acetic acid buffer pH 8.5, 0.2 mM EDTA, 500 μM NAD+ and 34 nM XDH in the absence or presence of oxypurinol. D, competitive inhibition of XOR by oxypurinol using xanthine as substrate. The reaction was followed at 550 nm in 3 ml of solution containing various concentration of xanthine, 0.1 M pyrophosphate/acetic acid buffer pH 8.5, 0.2 mM EDTA, 20 μM cytochrome c, 20 μM phenazine methosulfate and 2 nM XOR in the absence or presence of oxypurinol. [file mmc1.pdf]

Supporting Information

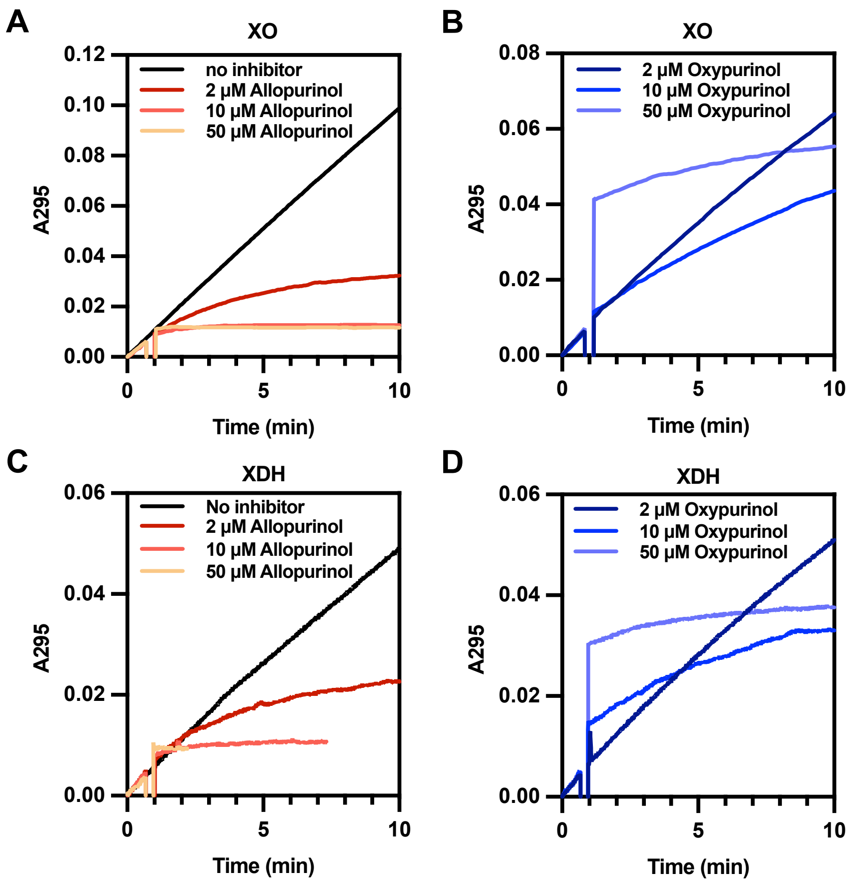

Figure S1 Time dependence of allopurinol and oxypurinol on XO and XDH.

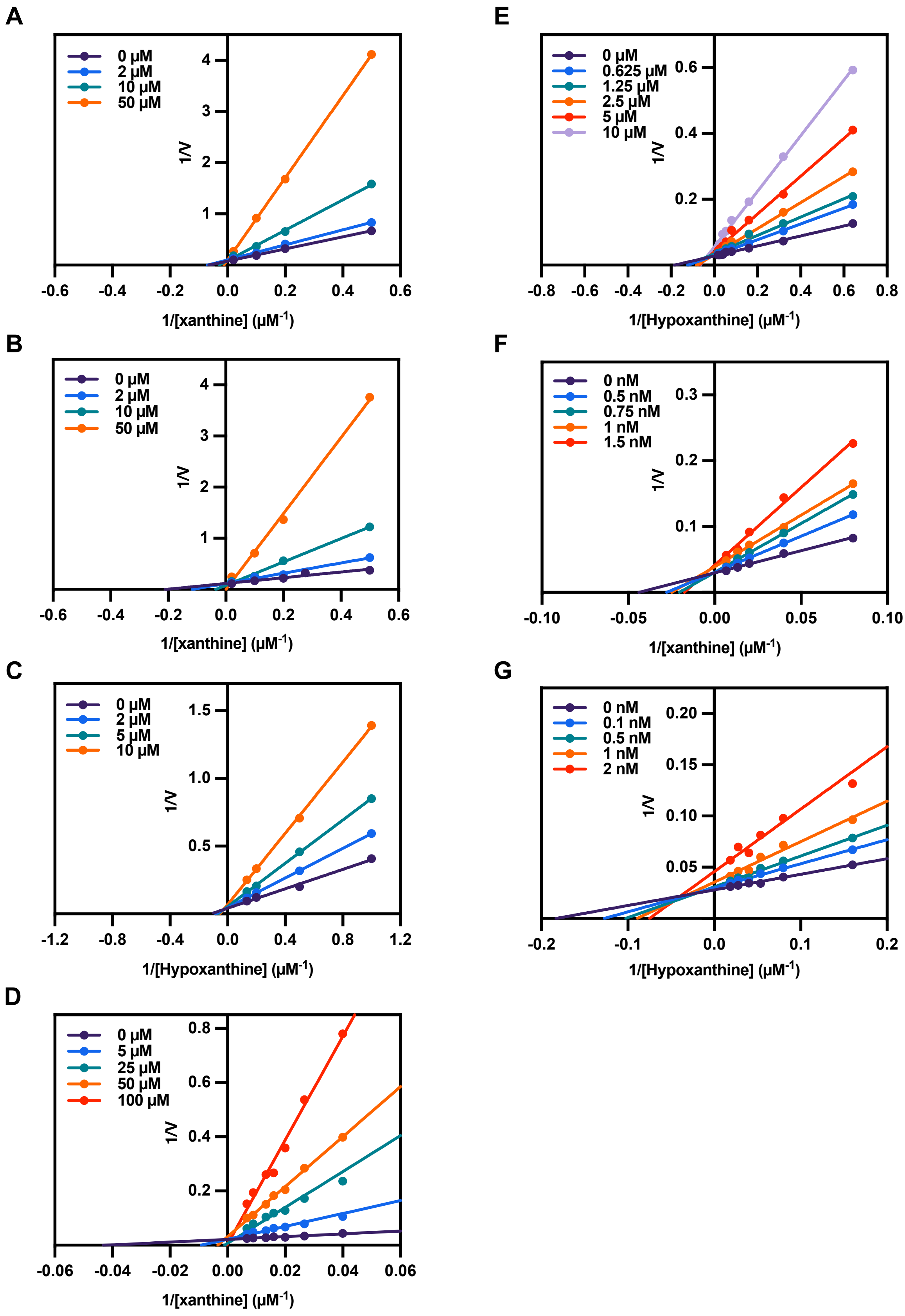

Figure S2 The XOR inhibition mode of oxypurinol and febuxostat.

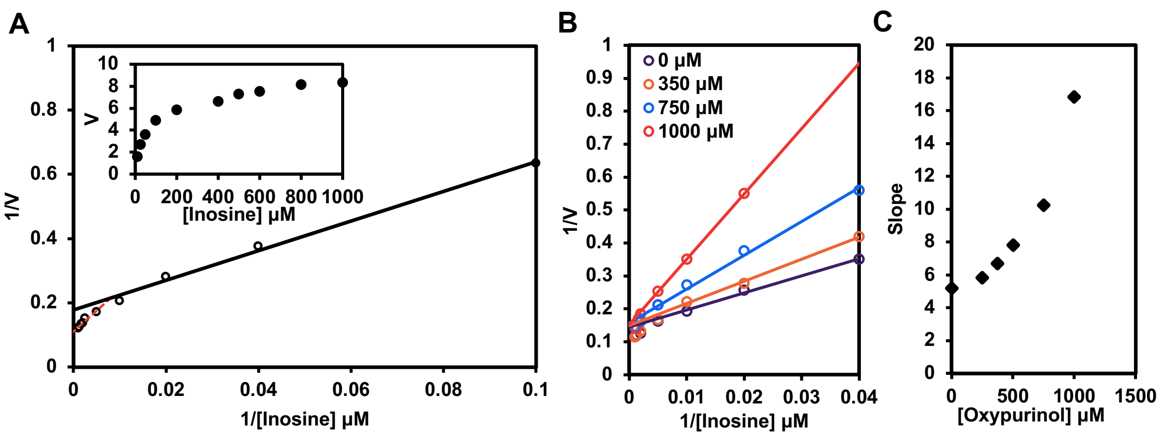

Figure S3 Allosteric inhibition of PNP by oxypurinol.

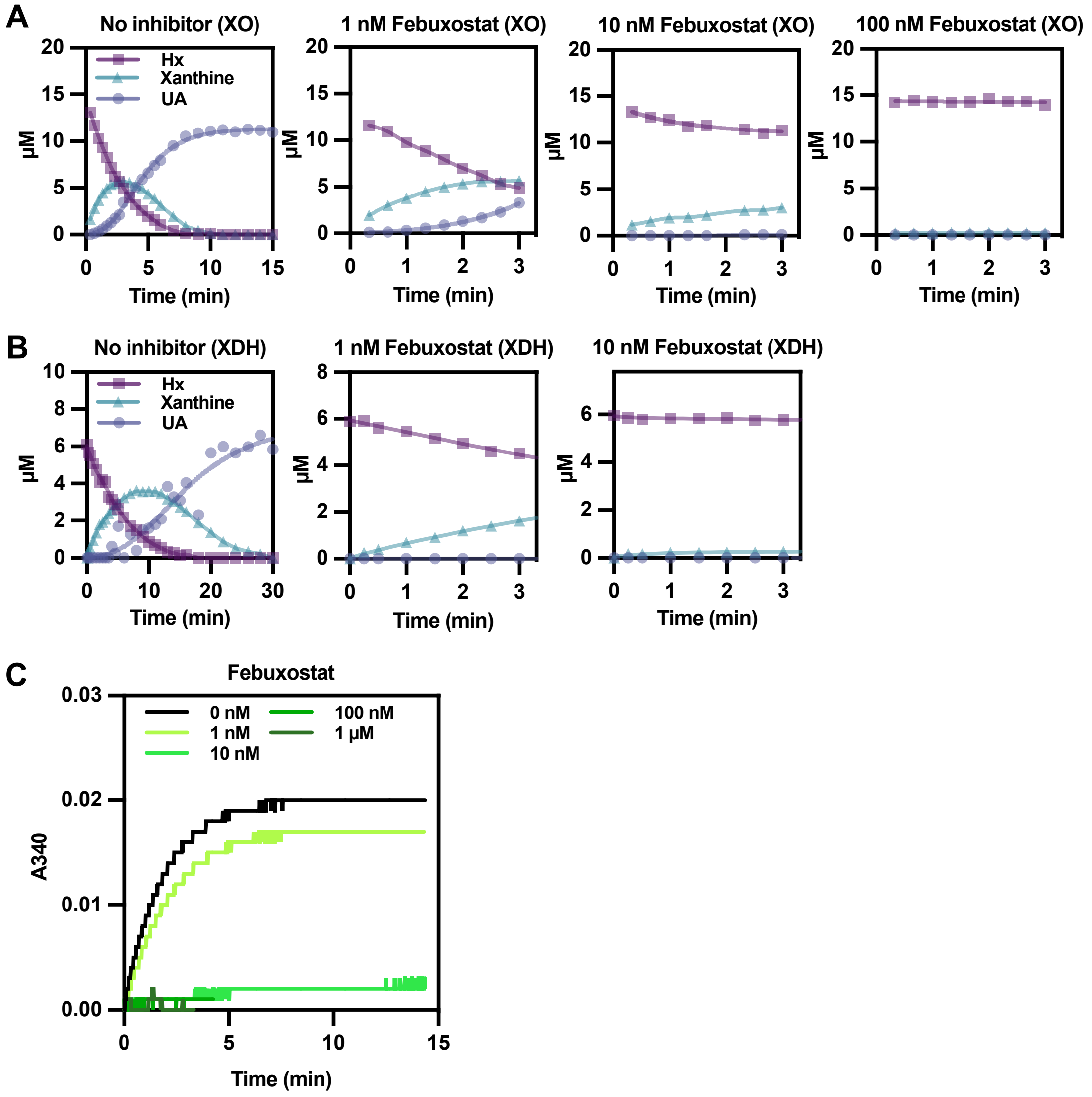

Figure S4 Inhibitory effects of febuxostat on hypoxanthine hydroxylation.
